# Comfort Needs of Renal Transplant Recipients: A Qualitative Analysis Guided by Kolcaba's Theory of Comfort

**DOI:** 10.1111/jocn.70188

**Published:** 2026-01-02

**Authors:** Cecília Carla Barroso Calazans, Jennara Cândido do Nascimento, Renan Alves da silva, Francisco Gilberto Fernandes Pereira, Lívia Moreira Barros, Joselany Áfio Caetano

**Affiliations:** ^1^ Federal University of Ceará Fortaleza Brazil; ^2^ University for International Integration of the Afro‐Brazilian Lusophony Redenção Ceará Brazil; ^3^ Federal University of Campina Grande Cajazeiras Paraíba Brazil; ^4^ Federal University of Piauí Teresina Piauí Brazil

**Keywords:** chronic kidney failure, kidney transplantation, nursing, patient comfort

## Abstract

**Aim:**

To analyse the comfort needs of patients following renal transplantation, guided by Kolcaba's Theory of Comfort.

**Design:**

A qualitative design was employed.

**Methods:**

This study was conducted at a Brazilian university hospital's renal transplant outpatient clinic. Forty‐six post‐transplant patients were purposively sampled by age, transplant time and clinic attendance. Face‐to‐face interviews were audio‐recorded, transcribed and conducted using a semi‐structured script. Data were analysed through thematic content analysis, guided by Kolcaba's Comfort Theory and relevant literature.

**Results:**

Participant narratives were categorised according to the contexts outlined by Kolcaba's Theory of Comfort: Physical, Environmental, Sociocultural and Psychospiritual. In the physical context, pain was identified as a major factor diminishing comfort after renal transplantation. In the environmental context, elements such as light, odour, sound, temperature and uncomfortable furnishings contributed to discomfort. In the sociocultural context, family support was highlighted as essential. In the psychospiritual context, religiosity played a key role in enhancing the comfort of transplant recipients.

**Conclusion:**

Spirituality, strengthened social support networks and non‐pharmacological comfort measures are essential for promoting comfort among patients following renal transplantation. These findings underscore the importance of integrated care approaches that address physical, emotional and social aspects to improve quality of life for this population.

**Implications for the Profession and/or Patient Care:**

Conceptual models in nursing provide a critical perspective for care and support the delivery of effective, evidence‐based interventions. By identifying the multidimensional comfort needs of post‐renal transplant patients, this study informs the development of targeted, holistic strategies for nursing and multidisciplinary practice in outpatient settings.

**Impact:**

This study examined the multidimensional comfort needs of post‐renal transplant patients and found that comfort is shaped by physical, environmental, sociocultural and psychospiritual factors. The results may guide global nursing and multidisciplinary outpatient care by informing integrated approaches that enhance the quality of life of transplant recipients.

**Reporting Method:**

This study was reported according to the COREQ framework.

**Patient or Public Contribution:**

No patient or public contribution.

## Summary

What does this paper contribute to the wider global clinical community?

- The study highlights the holistic comfort needs of post‐renal transplant patients, using Kolcaba's theory as a framework.
- Its findings can guide clinicians worldwide in developing comprehensive assessments and targeted interventions addressing the diverse factors that influence post‐transplant comfort.
- Furthermore, it underscores the universal need for holistic care approaches, recognising that post‐transplant comfort extends significantly beyond physiological management.

## 1 Introduction

Renal transplantation is the gold standard treatment for individuals with kidney failure requiring renal replacement therapy. This procedure offers significant advantages over dialysis, including improved health outcomes, reduced costs and prolonged survival (Chadban et al. 2020; Cunha and Lemos 2020). However, the post‐transplantation period presents numerous physical and emotional challenges that compromise patient comfort. Despite a successful transplant and a functioning graft, these individuals continue to manage a chronic condition that requires ongoing care. Consequently, it is crucial to recognise that this care is multifaceted, encompassing interrelated domains such as family relationships, social integration and interactions with healthcare services and professionals (Pedroso et al. 2020).

While previous research has explored various aspects of post‐transplantation recovery—including quality of life (Bagasha et al. 2020; Divdar et al. 2019; Finn; Malhotra 2019), self‐care (Song et al. 2022) and the therapeutic use of cannabis (Gitau et al. 2022)—a significant gap exists in the literature regarding the specific comfort needs of this population. Although many studies address related topics, they do not offer an in‐depth exploration of comfort. Therefore, understanding the stressful healthcare situations and unmet needs that extend beyond existing support systems is essential. This understanding is vital for planning and implementing nursing interventions designed to meet the comfort needs of post‐transplant patients, ultimately promoting their well‐being and integrity in this new phase of life.

The theoretical framework for this study is guided by Kolcaba's Comfort Theory. (Kolcaba 2003) defines comfort as ‘a fundamental experience of all human beings for relief, ease or transcendence arising from four contexts: physical, psychospiritual, social and environmental’ (p. 14). This concept is articulated through three states of comfort: (1) Relief: The state experienced when a specific comfort need has been met, (2) Ease: A state of calm, contentment or well‐being and (3) Transcendence: A state in which an individual can rise above problems or pain. Transcendence is considered the highest level of comfort, as it empowers individuals to plan their lives, solve problems and overcome stressful situations.

The concept of comfort pertains to holistic care, which may be experienced to varying degrees depending on factors involving individuals and their personal perceptions. Therefore, physical symptoms, environmental organisation, interpersonal relationships and individual beliefs and values are related to the patients’ experiences during care and their perception of comfort (Melo et al. 2017).

Historically, comfort has been linked to nursing care, presenting itself as a basic human need, regardless of whether an individual is experiencing an illness (Kolcaba 2003). In the context of chronic kidney disease, comfort is frequently compromised by procedures and routines associated with dialysis treatment and post‐transplant care. This can result in altered sleep patterns, anxiety, crying, discomfort, dissatisfaction with the situation, an inability to relax, restlessness, irritability, lamentation and fear, for example (Estridge et al. 2018; Melo et al. 2019).

Understanding comfort needs is crucial for developing nursing interventions that promote patient well‐being. Considering variables from the patient's perspective is decisive for creating best practices and policies aimed at ensuring the institutional integrity of kidney transplant services. This is essential to ensure behaviours that favour the pursuit of health and the institutional integrity of patients in a new phase of their lives. There is a scarcity of research addressing comfort as a dynamic and multidimensional phenomenon experienced by patients throughout their life and recovery trajectory, especially in the first few months. Therefore, this study aims to fill this gap by qualitatively exploring the comfort needs of post‐kidney transplant patients. In the present study, Kolcaba's Comfort Theory was used as a framework to analyse comfort needs, with a holistic understanding of care providing valuable insights for clinical practice, as it offers a robust conceptual framework for understanding these experiences in an integrated way.

Kolcaba's Comfort Theory allows for a deeper understanding of the lived experience, making it possible to capture the perceptions, feelings and meanings that recipients attribute to their recovery process—dimensions that are difficult to evidence through quantitative approaches. The theory helps identify non‐verbalised needs, such as fears, insecurities or adaptive challenges that directly impact comfort but are not captured by clinical indicators. It also promotes the personalization of care; by understanding how each patient perceives and prioritises their comfort, the healthcare team can design more patient‐centred nursing interventions. Finally, the theory integrates theory and practice by offering an analytical framework to classify and interpret needs, facilitating the translation of qualitative findings into measurable care actions.

Thus, using Kolcaba's Theory as an interpretive lens not only describes patient needs but also guides the development of holistic and well‐founded care strategies, contributing to improved therapeutic adherence, quality of life and clinical outcomes in the post‐transplant setting.

This study is guided by the central question: What are the comfort needs of patients following kidney transplantation? Accordingly, we aim to provide a qualitative, patient‐centred understanding of how these needs manifest during this critical period.

## 2 Methods

### 2.1 Design

This study employed an exploratory, descriptive qualitative design.

### 2.2 Theoretical Framework

The study was theoretically underpinned by Katharine Kolcaba's Theory of Comfort (Kolcaba 2003). This framework guided the development of the interview questions and the subsequent data analysis, particularly concerning the contexts in which comfort and discomfort are experienced.

The study's theoretical design involved the construction and validation of the data collection instrument. This process was conducted by three researchers with prior experience in studies on the topic of Comfort among individuals with chronic kidney disease, both in haemodialysis and renal transplant settings (Freire et al. 2020; Freire et al. 2021). Consequently, these researchers were instrumental in crafting the guiding questions for the semi‐structured interview guide, ensuring the relevance and appropriateness of the content investigated (Data S1).The guiding questions align with the structure of (Kolcaba 2003). Comfort Theory by exploring, respectively, the subjective aspects of comfort, discomfort within specific health contexts and the interventions, circumstances and/or experiences that promote relief, ease or transcendence.

Specifically, the first question addresses comfort from an individual perspective (the subjective dimension); the second identifies physical, psychosocial or environmental factors of discomfort post‐transplantation; and the third investigates health needs and strategies that contribute to well‐being. Together, these questions reflect the pursuit of comfort across the four contexts defined by Kolcaba: physical, psychospiritual, sociocultural and environmental.

### 2.3 Study Setting and Recruitment

Data were collected between April and May 2023 at the renal transplant outpatient clinic of a university hospital in Fortaleza, Ceará, Brazil. The qualitative research was conducted by a single researcher, a member of the Federal University of Ceará's Nephrology Nursing League. This individual, who was thoroughly trained and possessed expertise in the subject matter, conducted all interviews. This approach was chosen to ensure standardised application of the instrument, minimise interpretive bias and maintain methodological consistency throughout the data collection process.

No prior relationship was established with participants, nor were any researcher characteristics or information about the interviewers/facilitators disclosed before the study. This study followed the COREQ (Consolidated criteria for reporting qualitative research) guidelines for reporting (Tong et al. 2007) (Data S2).

### 2.4 Inclusion and Exclusion Criteria

Forty‐six renal transplant patients were recruited using purposive sampling. Participants were eligible if they were 18 years or older, had undergone a single kidney transplant, were in outpatient follow‐up for 3 months to 1 year, were clinically stable and able to communicate verbally. Individuals who had undergone dual transplants, experienced graft loss or were 10 or more years post‐transplantation were excluded to minimise potential bias. This specific post‐transplant timeframe was chosen as it captures the critical transition from hospitalisation to home recovery and initial outpatient follow‐up, periods relevant to the manifestation of comfort needs.

### 2.5 Participant Selection

Data saturation guided the determination of sample size, achieved after 46 interviews. Saturation occurred when no new insights emerged from additional data, indicating redundancy. As per (Bardin 1977), this point was recognised during categorization and analysis, confirming that further interviews would not yield new elements.

Participants were recruited through convenience sampling at the renal transplant outpatient clinic. During the data collection period, the researcher responsible for conducting the interviews, with the support of the local healthcare staff, directly approached eligible patients while they awaited their routine consultation. Following a brief presentation of the study and its objectives, interested individuals were invited to a private room where the informed consent form was read and discussed.

### 2.6 Data Collection

Data were collected using semi‐structured interviews guided by an instrument developed by the authors. The guide was divided into three parts: (1) Sociodemographic data: Sex, age group, place of birth, educational level, marital status, religion and family income, (2) Clinical data: Time since transplantation, complications during surgery and adherence to the medication regimen and (3) Comfort experiences: Guiding questions based on Kolcaba's Theory of Comfort, designed to explore the physical, environmental, sociocultural and psychospiritual contexts in which comfort and discomfort were experienced following renal transplantation. The interviews were audio‐recorded and transcribed, but transcripts were not returned to participants for comment.

Interviews were scheduled on 3 days per week to align with the operational flow of the renal transplant outpatient clinic. Each participant was interviewed on a single occasion. The interviews were conducted between 8 a.m. and 5 p.m., taking place either during the intervals between or at the conclusion of their outpatient appointments. Preference was given to days with the highest patient volume. Each participant was interviewed individually in a reserved space for a maximum of 60 min.

During the initial coding process and the identification of emergent themes from the interviews, we identified significant elements that did not readily align with the pre‐established analytical framework. Recognising the relevance of this data for understanding the comfort experience in the investigated context, the decision was made to deepen the analysis by returning to the field. The objective of this additional phase was to understand in greater depth how aspects such as income, social support and religiosity were intertwined with the participants’ lived perception of comfort. This strategy enhanced the interpretive depth of the findings.

### 2.7 Trustworthiness and Rigour

Based on the theoretical framework, three core questions were posed: (1) How do you perceive the concept of ‘comfort’ in your daily life?, (2) Describe the feelings or situations that have caused you discomfort since your kidney transplant and (3) What would you identify as your primary health needs after the kidney transplant? In what ways did addressing those needs help you feel more comfortable during this period? The interview questions were pilot‐tested with three transplant recipients who were not part of the study sample. This was done to evaluate aspects such as credibility, transferability, dependability, confirmability and reflexivity.

Sociodemographic and clinical data were collected first, followed by the semi‐structured interview component. Each participant was interviewed individually in a private setting, with interviews lasting up to 60 min. Before commencing the interview, the researcher presented the study objectives, benefits and potential risks. Participants provided their agreement and signed the consent form after all clarifications were made. Interviews ceased when the researcher determined that theoretical saturation had been reached, indicated by the repetition of information in participant accounts. No repeat interviews were conducted.

To ensure the rigour and trustworthiness of the research findings, several methodological strategies were adopted throughout the study's development. During the data organisation and analysis process, we conducted peer debriefing sessions to obtain an external critical perspective, which helped identify potential biases or inconsistencies in the different stages of the investigation. We maintained detailed records of all study phases—including methodological decisions, data collection and analysis—which promotes the transparency of the process and allows for potential auditability. Furthermore, Kolcaba's theoretical framework, which underpins the understanding of comfort in this study, was continuously revisited throughout the research process to ensure theoretical‐methodological coherence and to consistently guide the interpretation of the results.

### 2.8 Data Analysis

**FIGURE 1:**
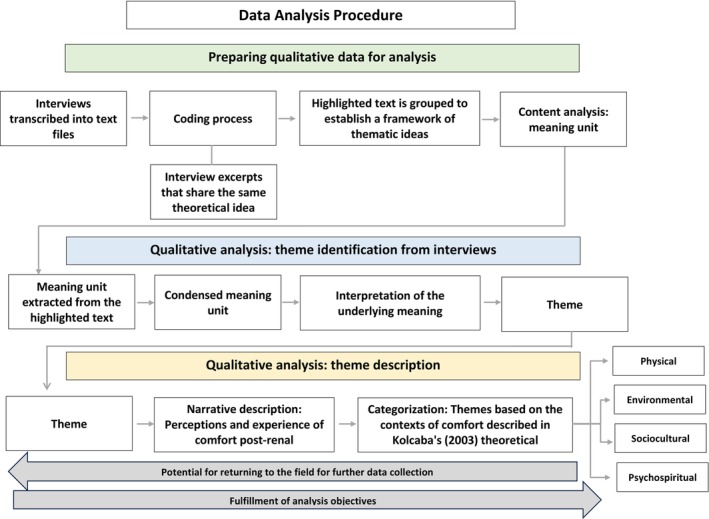
Data analysis procedure. ++Adapted from (Polit and Beck 2018). [Colour figure can be viewed at wileyonlinelibrary.com]

The interview transcripts were organised and analysed using thematic content analysis as proposed by (Bardin 1977). This process involved three distinct stages: (1) Pre‐analysis: Data were initially organised by transcribing all interviews verbatim. This was followed by immersion reading of the entire dataset to gain a general understanding of the content, (2) Material exploration: Transcribed narratives were segmented into units of analysis (meaning units), which were then coded and labelled according to the concepts they represented. Coded material was subsequently grouped based on shared concepts, forming initial themes and (3) Treatment and interpretation: In the final stage, the categorised data were interpreted through the lens of (Kolcaba 2003) Theory of Comfort and relevant literature (Figure 1). Findings were structured according to the theory's contexts of (dis)comfort: physical, environmental, sociocultural and psychospiritual.

Each analysis phase was conducted independently by two researchers, with a third consulted in case of discrepancies. Through analysis and discussion, the researchers reached full agreement on the results.

### 2.9 Reporting

Participant quotations were used to illustrate the themes and findings, with each quotation identified by a unique participant number. The data presented were consistent with the findings and major themes were clearly articulated. Diverse cases and minor themes were also discussed to provide a comprehensive understanding of the data.

### 2.10 Ethical Considerations

This study was conducted in adherence to institutional ethical guidelines for human research. The research project received approval from the Institutional Review Board under protocol number 6.066.577/2023. To ensure participant anonymity, pseudonyms (P1, P2–P46) were assigned in the interview transcriptions using Microsoft Word. All participants provided written informed consent prior to data collection.

## 3 Results

### 3.1 Participant Characteristics

A total of 46 renal transplant recipients participated in the study. The sample consisted of a slight majority of women (52.2%), with a mean age of 47.76 years. Further sociodemographic and clinical details are presented in Table 1.

**TABLE 1:** Sociodemographic and clinical characterisation of renal transplant patients in outpatient follow‐up.

Variables	*n* (%)	95% CI	Mean (95% CI)
Sex			
Male	22 (47.8)	(33.9–62.0)	
Female	24 (52.2)	(38.0–66.1)	
Age (years)			47.76 (43.60–51.92)
18–24	1 (2.2)	(0.2–9.7)	
25–30	5 (10.9)	(4.3–22.2)	
31–39	7 (15.2)	(7.1–27.6)	
40–49	13 (28.3)	(16.9–42.3)	
50–59	10 (21.7)	(11.8–35.1)	
60–69	6 (13.0)	(5.6–24.9)	
70–79	4 (8.7)	(3.0–19.4)	
80–89	0 (0.0)		
Place of origin			
State capital	28 (60.9)	(46.5–74.0)	
Countryside	18 (39.1)	(26.0–53.5)	
Education level			
Illiterate	4 (9.1)	(3.1–20.2)	
Able to read and write	4 (9.1)	(3.1–20.2)	
Incomplete elementary school	2 (4.5)	(1.0–13.8)	
Completed elementary school	7 (15.9)	(7.4–28.7)	
Incomplete high school	10 (22.7)	(12.3–36.6)	
Completed elementary school	10 (22.7)	(12.3–36.6)	
Higher education	7 (15.9)	(7.4–28.7)	
Marital status			
Single	14 (30.4)	(18.6–44.6)	
Married/cohabiting	18 (39.1)	(26.0–53.5)	
Divorced	9 (19.6)	(10.1–32.7)	
Widowed	5 (10.9)	(4.3–22.2)	
Religion			
Catholic	23 (50.0)	(35.9–64.1)	
Protestant	10 (21.7)	(11.8–35.1)	
Agnostic	8 (17.4)	(8.6–30.2)	
Other religions	5 (10.9)	(4.3–22.2)	
Family income (BRL)			
Up–500	3 (6.5)	(1.9–16.4)	
500–1000	6 (13.0)	(5.6–24.9)	
1000–1500	16 (34.8)	(22.3–49.1)	
1500–2000	14 (30.4)	(18.6–44.6)	
Above 2000	7 (15.2)	(7.1–27.6)	
Time since surgery			
3–6 months	10 (21.7)	(11.8–35.1)	
7–11 months	13 (28.3)	(16.9–42.3)	
1 year	23 (50.0)	(35.9–64.1)	
Surgical complications			
Yes	12 (26.1)	(15.1–40.0)	
No	34 (73.9)	(60.0–84.9)	
Difficulty with medication adherence			
Yes	15 (32.6)	(20.4–46.9)	
No	31 (67.4)	(53.1–79.6)	

### 3.2 Comfort Needs

Analysis of participant accounts revealed comfort needs across the four contexts outlined by Kolcaba's Theory of Comfort: Physical, Environmental, Sociocultural and Psychospiritual. Within each context, thematic analysis identified key subcategories, which are detailed below, alongside illustrative participant quotes. Twelve sub‐themes were identified and categorised into four main themes.

Within the Physical context, post‐operative pain emerged as the primary source of discomfort, frequently linked to invasive procedures and infectious complications. The narratives demonstrate that patient comfort following renal transplantation is a multifaceted phenomenon that changes over time and is conditioned by physical, emotional, social and spiritual factors. This understanding broadens the perspective of nursing and the healthcare team regarding care, emphasising that promoting comfort extends beyond mitigating physical symptoms, requiring strategies for humanization, emotional support and the creation of therapeutic environments that foster integral well‐being.

I was sore at the beginning, because I had those things, you know! (referring to catheters, drains and the central venous line in the jugular), but then it passed. Thank God I didn't feel any more discomfort. [P45]

In the beginning, I had to be hospitalized again, I almost lost the kidney, I had several biopsies, I was punctured several times. [P37]

After leaving the operating room, everything hurts. Until the third month, it's discomfort. [P36]

The accounts highlight that the immediate post‐operative period is characterised by intense pain, the presence of invasive devices, the risk of complications and prolonged hospitalizations. This experience translates into a momentarily absent relief, as the body adapts to the graft and faces the challenges inherent to the surgery. However, the pain and physical suffering are narrated as transitory. In this sense, the initial discomfort can be interpreted as part of the transition process from illness to rehabilitation, where the patient recognises the finitude of the physical suffering and values the progressive improvement.

Strategies aimed at relief included preventive measures against infection and adherence to the prescribed medication regimen, although some participants reported difficulties managing medication schedules.

I always leave the house putting on sunscreen, wearing my mask and using alcohol gel on my hands. [P5]

I avoid places with lots of people because my immunity is low. I avoid contact with sick people, I always keep my distance. [A37]

Patients expressed concerns related to personal hygiene, infection prevention, the use of masks, hand hygiene with alcohol‐based gel and adherence to the immunosuppressive regimen. Medication management is viewed as central to graft and health maintenance, highlighting strategies such as cell phone alarms and visual reminders affixed in strategic locations around the house to assist the transplant recipient in adhering to the proposed treatment.

For the medication, I followed the booklet the pharmacist gave me and I also set my phone alarm to remember the time at first, but that was only at the beginning. [P19]

The pharmacist gives you the names of the medications with the times on another paper. That helped me a lot, because I put it right on the fridge so I wouldn't forget, just like a magnet! Wow. That made all the difference, because sometimes I forgot the medication time. I won't lie, right?. [P33]

**FIGURE 2:**
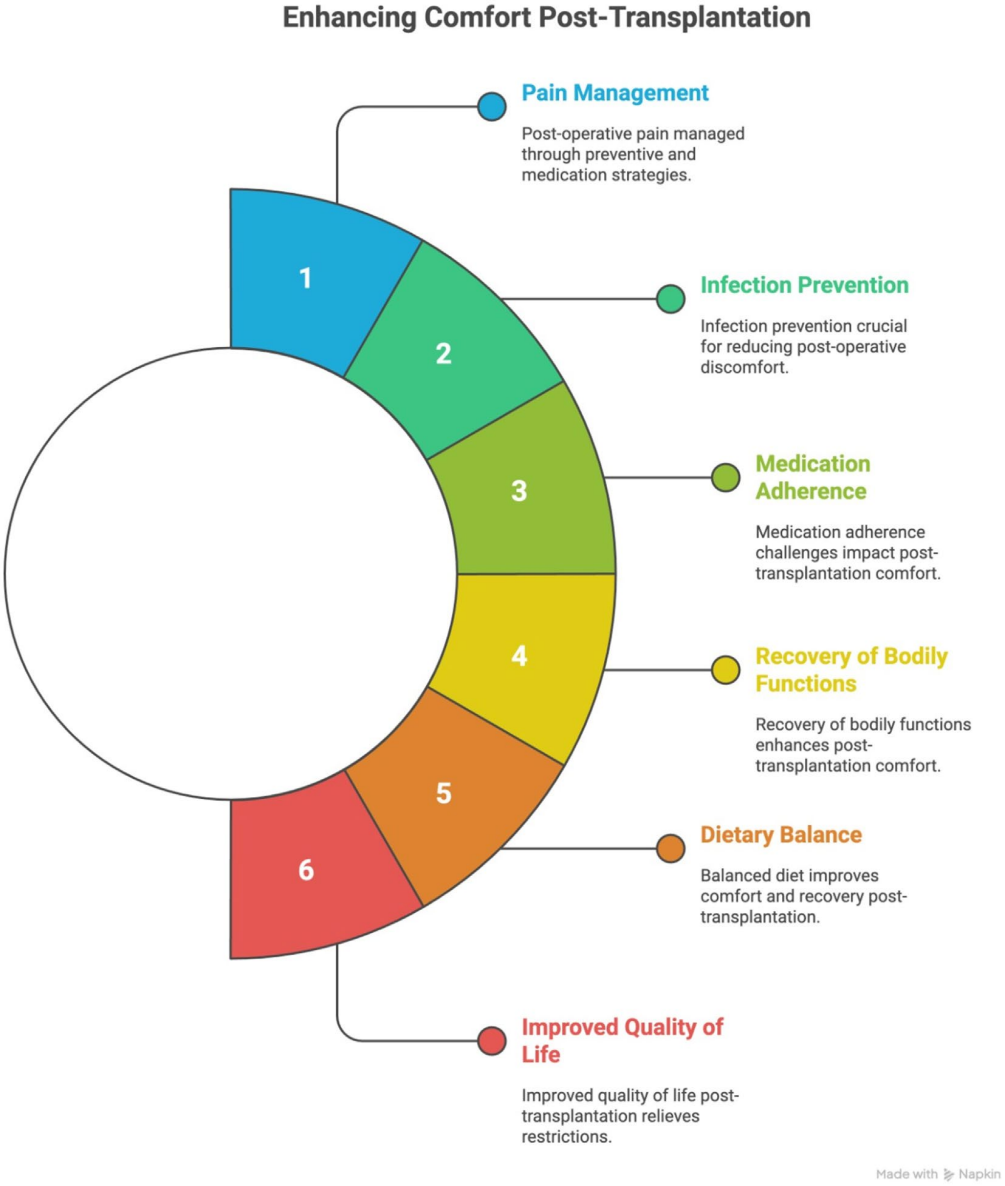
Interconnected factors influencing the comfort of patients following transplantation. [Colour figure can be viewed at wileyonlinelibrary.com]

Here emerges the comfort associated with safety, in which the patient perceives the need to act actively to protect the graft. Nursing and the multiprofessional team appear, implicitly, as facilitators, by providing handouts, guidance and support for the management of the therapeutic regimen. Figure 2 provides a visual breakdown of post‐transplant comfort factors.

Conversely, the recovery of bodily functions (such as urine production) and the ability to maintain a balanced diet were noted as significantly enhancing comfort following transplantation.

Comfort, for me, is being off the machine, being able to drink water and pee, things I couldn't do anymore. [P44]

The newly transplanted patient has to have a very balanced diet, drink lots of water so the kidney works even better!. [P2]

My main need now is to eat well, drink more water so I don't lose the kidney. [P4]

The statements also reveal that, following transplantation, comfort is related to the possibility of resuming a more varied and healthy diet. Eating without as many restrictions, ingesting water and eliminating urine are described as symbolic daily conquests, representing liberation from the deprivations imposed by chronic kidney disease and haemodialysis. This dimension of comfort transcends mere physical satisfaction and reflects the reconstruction of autonomy and quality of life.

My comfort improved after the transplant, life is totally different. With that chair and the time on haemodialysis, I felt uncomfortable, I had a lot of cramping, nausea during it, sometimes my blood pressure would drop too. [P6]

After I was transplanted my life improved 100%, because the transplant improves our comfort, as you get rid of the machine and can do your own things freely. [P2]

The transplant was the best thing that happened in my [life], because I couldn't leave the house because I felt so bad when I was doing haemodialysis. [P29]

Walking, getting some sun, I've already been to the beach once since the surgery. [P26]

**FIGURE 3:**
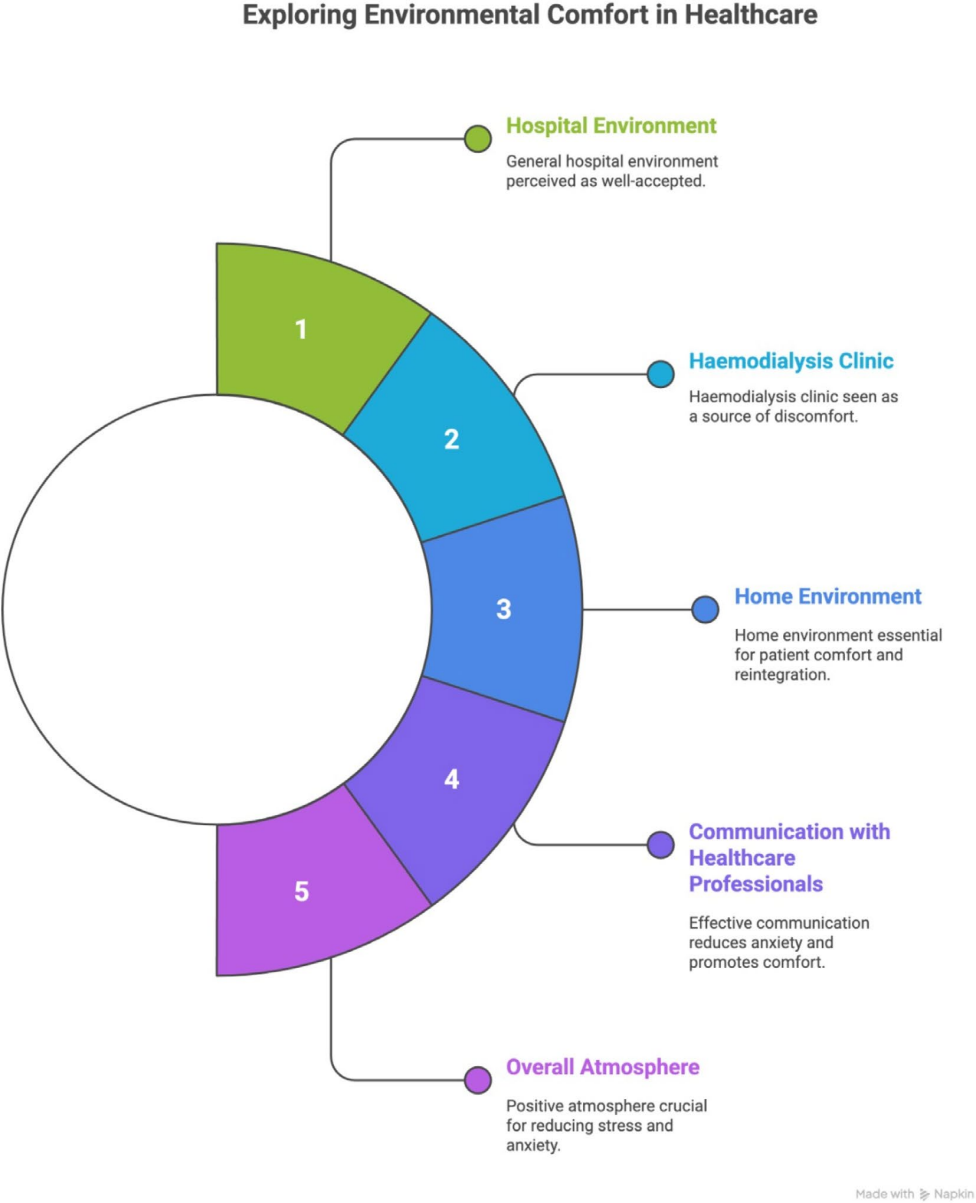
Key interventions aimed at enhancing comfort in post‐transplant patients. [Colour figure can be viewed at wileyonlinelibrary.com]

The following infographic summarises the key findings of the investigated context (Figure 3).

Within the Environmental context, findings revealed that although the general hospital environment was well‐accepted, the haemodialysis clinic was perceived as a source of significant discomfort. This was attributed to factors such as inadequate furnishings, excessive noise, unpleasant odours and poor lighting. In contrast, being at home was considered essential for achieving comfort, underscoring the positive impact of transplantation on patients’ reintegration into daily routines and family life. The home symbolises freedom, privacy, autonomy and belonging—values that haemodialysis tends to undermine.

Comfort is being in a pleasant environment, smelling nice, a good sofa! A spacious environment. [P22]

Comfort is being in a place that's healthy, a pleasant environment, cool, no heat, right! Comfort is that. [P16]

For me, comfort is this: getting off those machines and being well at home with the family. Having that comfort, right!?. [P38]

Not being on that machine anymore, that's already a big deal, because it's a tough routine! You have to be there at least three times a week. [P5]

After I was transplanted my life improved 100%, because the transplant improves our comfort, as you get rid of the machine and can do your own things freely. [P2]

The comparison between the haemodialysis period and life after transplantation is recurrent. Terms such as ‘getting rid of the machine’, ‘doing your things freely’, ‘leaving the house’ and ‘being able to walk again, sunbathe, go to the beach’ express that comfort is not only physical, but also existential and psychospiritual.

The experience of dialysis is described as imprisoning, exhausting and discouraging, whereas transplantation is perceived as a rebirth. This experience connects to the dimension of transcendence, where the patient re‐signifies their life, values freedom and associates the healing process with gratitude towards God and medicine.

**FIGURE 4:**
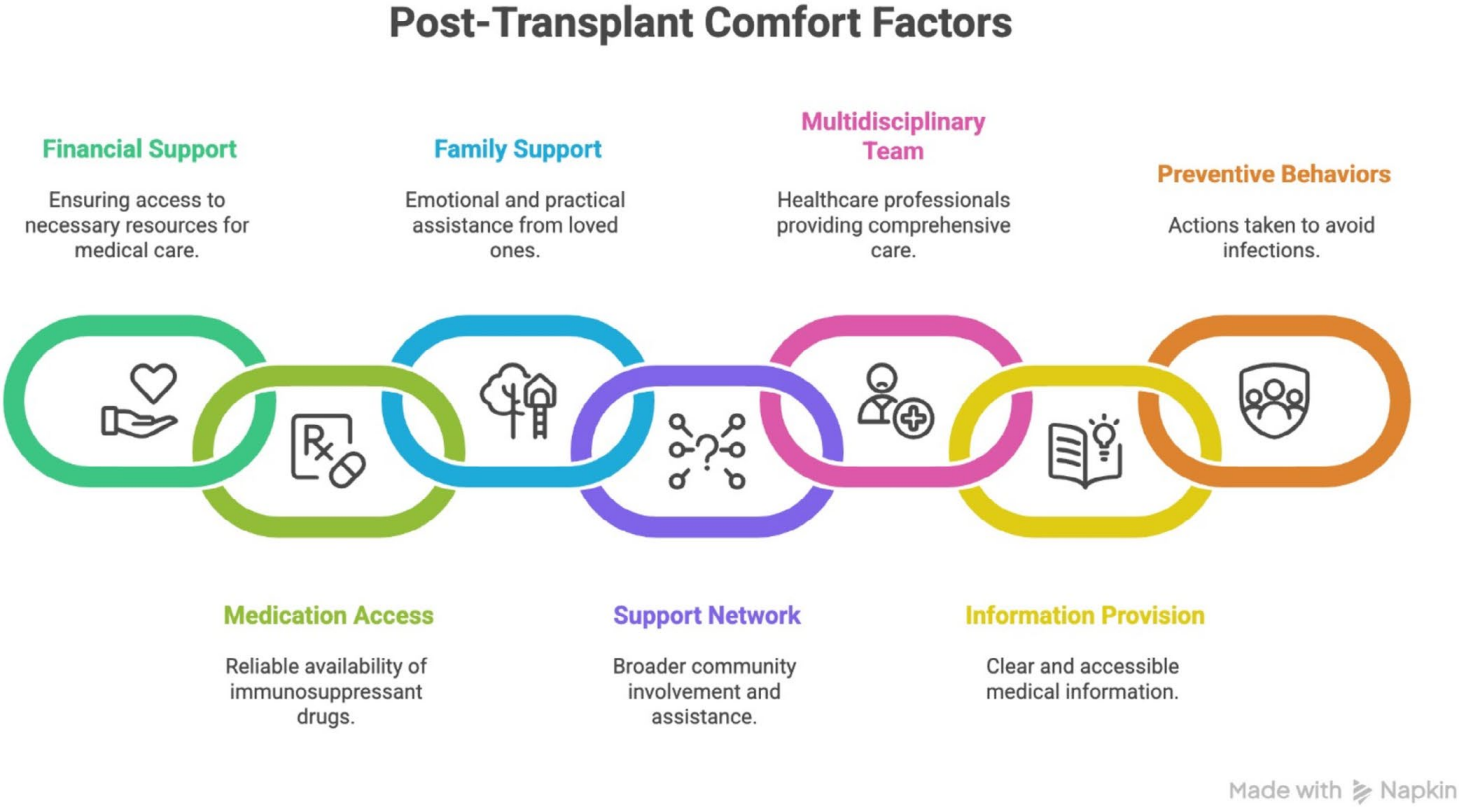
Key domains of environmental comfort in the post‐transplantation period. [Colour figure can be viewed at wileyonlinelibrary.com]

The following infographic illustrates further details of the investigation concerning the environmental comfort (Figure 4).

Within the Sociocultural context, financial support and reliable access to immunosuppressant medications were identified as crucial factors impacting recipient comfort. Family support and the presence of a broader support network were highly valued, positively influencing adaptation to the post‐transplant lifestyle. The following interview excerpts highlight the economic challenges faced after a kidney transplant, as well as the importance of support from family and/or other significant individuals.

Comfort is being in a pleasant environment, smelling nice, a good sofa! A spacious environment. [P22]

Comfort is being in a place that's healthy, a pleasant environment, cool, no heat, right! Comfort is that. [P16]

For me, comfort is this: getting off those machines and being well at home with the family. Having that comfort, right!?. [P38]

Not being on that machine anymore, that's already a big deal, because it's a tough routine! You have to be there at least three times a week. [P5]

After I was transplanted my life improved 100%, because the transplant improves our comfort, as you get rid of the machine and can do your own things freely. [P2]

The relationship with the multidisciplinary team also emerged as essential, particularly concerning the provision of information and supportive care, which enhanced feelings of safety and well‐being.

The doctors’ treatment, the nurses’ welcome, that counts a lot towards comfort. [P37]

I think having correct information about what is really happening to me, because I need to know regardless of my health status. [P35]

Being in a place where I feel good, surrounded by people who like me. I feel comfortable. [P17]

The treatment received from the multiprofessional team, especially physicians and nurses, was identified as an essential factor for the perception of comfort. Welcoming (or Receptivity), listening and respect emerged as valued practices. Furthermore, the provision of correct and clear information regarding the disease process and treatment was understood as promoting safety and tranquillity.

This dimension reinforces the relevance of therapeutic communication and the humanization of care, which are pillars of nursing practice. Feeling well treated and having correct information about one's own health status are dimensions that produce safety, confidence and a sense of integral care. This aspect conveys the importance of human relationships in coping with illness, aligning with what nursing understands as patient‐centred care.

Finally, within the Psychospiritual context, emotional adaptation proved challenging for participants, particularly during the initial post‐transplantation period, with common reports of distress, dissatisfaction and negative thoughts regarding graft integrity and their overall health status during this period.

After the transplant, at 3 months, I got a urinary tract infection Then it was very uncomfortable, because I had to have a catheter put in, I had to spend 14 days taking medication. That was very uncomfortable, one of the worst things!. [P26]

During that period I went through serious family problems that influenced my treatment. I also have some very down thoughts (I won't lie) Not every day am I okay, 100%, but I do my things and keep going. [P33]

Well, your psychological state gets a bit shaken at the beginning, right, because you have constant appointments, you're still getting to know what the transplant is all about. [P27]

The biggest discomfort is going through a treatment like this, taking a ton of medicines and not being cured, because that's the truth: you're not cured!!. [P1]

Spirituality and faith were frequently highlighted as key sources of emotional strength, aiding patients in coping with uncertainties and fostering resilience amidst the changes encountered.

Faith, for me, is everything (it's more than health). If you have faith, you have health. [P37]

And I give thanks every day for my transplant. Only wonder really, only good things really. God's work!!. [P42]

Being okay with yourself and always having faith in God so things don't go wrong. [P19]

Being transplanted was everything I wanted, I asked God for it every day. [P15]

Furthermore, mental health emerged as a clear priority, with participants valuing self‐care strategies such as psychological counselling and activities designed to promote emotional equilibrium.

Doing something to occupy the mind and not be alone. Work more on mental health!. [P19]

And sometimes a little sadness hits, but you always have to be firm and strong to feel really comfortable!. [P19]

Taking care of my mental health (especially, laughs). Now in the beginning it's a lot of change. So you have to be prepared for these changes. [P46]

Taking care of my mental health! I want to go back to the psychologist, because some days I'm very anxious. [P26]

These findings highlight that renal transplantation significantly impacts patients’ perception of comfort, reinforcing the importance of holistic care approaches. Such approaches must consider not only physiological aspects but also environmental, sociocultural, and psychospiritual factors to effectively promote overall well‐being.

Negative experiences, such as pain, post‐transplant complications and anxiety, coexist with coping strategies founded on faith, social support and mental health care. This analysis reinforces the need for person‐centred nursing interventions that consider not only the biomedical aspects of treatment but also the emotional, social and spiritual dimensions, thereby promoting integral and humanised care.

**FIGURE 5:**
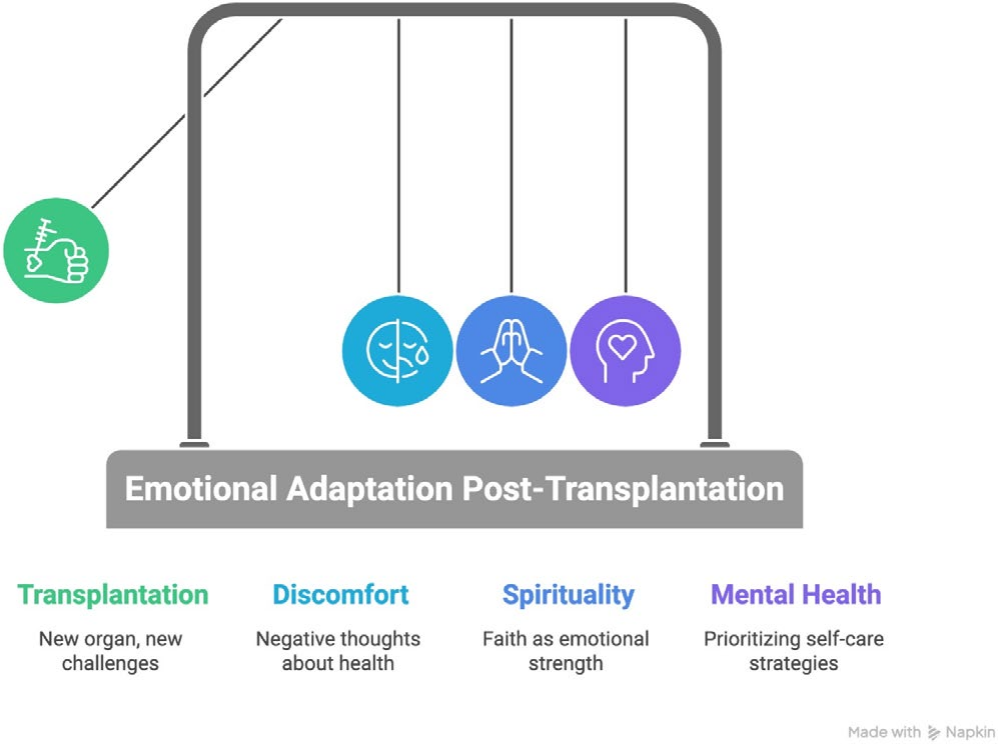
The process of emotional adaptation in the post‐transplantation period. [Colour figure can be viewed at wileyonlinelibrary.com]

Finally, the following infographic presents a comprehensive overview of the results concerning emotional adaptation post‐transplantation (Figure 5).

## 4 Discussion

This study explored the comfort needs of patients following renal transplantation, highlighting pain as the primary source of physical discomfort, particularly in the immediate post‐operative period, consistent with previous findings (Desantana et al. 2020; Lambourg et al. 2021). Post‐operative pain is a common occurrence after kidney transplantation, with its highest frequency reported in the initial months following surgery. The prevalence of pain among renal transplant recipients is estimated at 46%, although there is no consensus on this figure in the literature (Fleishman et al., 2020; Lambourg et al. 2021).

The use of invasive devices and biopsies also contributed to this painful experience, aligning with other research (Cordeiro 2021; Schnuelle 2023). Furthermore, the requisite immunosuppression increases vulnerability to infection, underscoring the essential nature of preventive measures (Cardoso et al. 2024).

Thus, the body of narratives allows us to state that the comfort of the chronic renal transplant patient is a dynamic and relational process that transitions between pain, relief, adaptation, safety and transcendence. Initially marked by physical discomforts, it expands to include social and spiritual dimensions, demonstrating that the notion of comfort cannot be reduced to the absence of pain but involves integral well‐being, the resumption of autonomy and a feeling of freedom regarding life.

Adherence to the treatment regimen was recognised as fundamental for graft survival; however, participants reported challenges including forgetting doses, managing side effects and medication handling difficulties, echoing previous studies (Santos et al. 2017; Silva et al. 2022). Poor therapeutic adherence is linked to adverse outcomes like rehospitalization, the need for dialysis and increased morbidity and mortality, all of which drive up healthcare costs. This makes it a significant public health issue (Bello et al. 2022).

The quality of life for transplant recipients is notably higher compared to their time on dialysis, primarily due to greater freedom in daily activities, previously restricted by dialysis sessions. Thus, kidney transplantation improves the physical, mental and social well‐being of those who undergo the procedure (Camelo et al. 2021).

Thus, comfort is intrinsically related to the patient's autonomy, who seeks to participate actively in the treatment as a way to preserve well‐being and quality of life, even when faced with the limitations imposed by the illness.

Haemodialysis sessions often limit patients’ ability to engage in social, intellectual and physical activities, leading to a reduced quality of life (Kułakowska et al. 2023). Research indicates that kidney transplant recipients perceive haemodialysis as a difficult process due to dietary restrictions, strict fluid control, polypharmacy and unpleasant symptoms like pain, muscle weakness, depression and anxiety. Consequently, individuals often experience feelings of confinement and a decline in their ability to perform activities (Colombijn et al. 2021; Ribeiro et al. 2021).

Therefore, there is a central dichotomy between the haemodialysis machine and life outside of it. For many participants, ‘no longer being on that machine’ represents a milestone of relief and freedom, given that the three‐times‐a‐week dependence on the procedure is experienced as a heavy routine that removes autonomy, restricts time and limits leisure and social activities. In this sense, renal transplantation emerges as the ultimate symbol of comfort, as it allows for the resumption of life ‘freely’, breaking the condition of imprisonment to the machine.

Sociocultural factors revealed that kidney transplant recipients often faced significant economic challenges. Their incomes were frequently insufficient to cover treatment costs and travel expenses for appointments, aligning with previous findings (Campos et al. 2017). Despite these constraints, financial assistance from family members helped participants cope with difficulties (Santos et al. 2020).

The need for frequent travel/displacement results in not only high costs but also emotional repercussions, such as feelings of sadness and frustration due to treatment dependency. Thus, comfort is manifested in the possibility of moving with dignity and without financial burden—a dimension scarcely considered by traditional biomedical models, despite its direct influence on therapeutic adherence.

Support from family and significant others was also essential for post‐transplant adaptation, providing both emotional buffering and practical self‐care assistance (Borges et al. 2016; Lopes et al. 2020). Therefore, the family and the support network play an essential role in promoting comfort, as the domestic environment and affective ties provide security, welcoming (or support) and a sense of normality in the face of chronicity. These findings reinforce that comfort constitutes a relational and contextual construct, sustained by social and emotional interactions and not a strictly individual phenomenon.

A meta‐analysis of Chinese studies on social support among kidney transplant recipients corroborates these findings, indicating that family and social support are crucial for rehabilitation. This support provides psychological relief and thus, emotional backing can be leveraged to channel negative emotions in these patients.

Within the psychospiritual domain, participants expressed varied perceptions of the transplant experience, ranging from feelings of overcoming adversity, gratitude and faith to emotions like distress and insecurity, reflecting the complex nature of this journey (Lisieski and Caviquioli 2020). After kidney transplantation, participants identifying as Christian reported higher comfort levels compared to those self‐identifying as Catholic or adhering to other beliefs such as spiritualism. This difference may stem from the more effective support networks often provided by Christian communities, which assisted individuals in coping with post‐transplant challenges, including help with transportation to follow‐up appointments and/or financial aid during the perioperative period. Many transplant recipients, often primary household providers, had to temporarily cease formal or informal activities to adhere to treatment.

Therefore, having a religion influences coping with illness and overall quality of life. Spirituality, faith and religious beliefs can actively aid in the health‐disease process, promote healthy habits, facilitate adaptation to illness and help prevent complications (Cruz et al. 2017). During this adaptation process, special attention to the transplant recipient's mental health is crucial, as it involves significant emotional challenges and constant adjustments. To better manage these circumstances, interviewees reported employing strategies such as psychological counselling and self‐care practices to promote well‐being and resilience.

Therefore, it is concluded that comfort in chronic renal patients is a multidimensional phenomenon that integrates physical and environmental aspects, which relate to the body and the treatment space; relational and communicational aspects, involving welcoming and access to information; emotional and social aspects, which are linked to social interaction and the support of significant others; and finally, subjective and existential aspects, which are marked by the search for autonomy and freedom in relation to the treatment.

## 5 Strengths and Limitations

A key strength of this study lies in its use of Kolcaba's Theory of Comfort, providing a robust, holistic framework to explore the multifaceted comfort needs of patients following renal transplantation. The qualitative methodology facilitated an in‐depth understanding of participants’ lived experiences during a critical adaptation period (3 months to 1 year post‐transplant), achieving theoretical saturation with a sample of 46 participants.

Study limitations included data collection at a single reference transplant centre, which may restrict the diversity of experiences and contexts reported by participants. The relatively short period for establishing a solid therapeutic bond could also have impacted the quality of responses, as they might have been influenced by subjective and emotional aspects at the time of the interview, posing a challenge for standardised interpretations.

Despite these limitations, the findings presented here provide important insights into understanding comfort needs in the context of kidney transplantation. This understanding can inform the development of interventions better tailored to the genuine needs of these patients.

## 6 Conclusions

This study advances the current understanding of patient comfort by revealing that for post‐renal transplant individuals, it is shaped by subjective and sociocultural factors, notably religiosity and medication adherence. We found that active participation in religious communities, especially among protestant church members, provides a social support network that strengthens feelings of physical and spiritual comfort post‐transplantation. Moreover, despite a complex therapeutic regimen and invasive procedures, all participants reported a superior level of comfort when compared to their prior haemodialysis experience. These findings expand the concept of comfort in renal care, incorporating dimensions seldom explored in the literature, such as the role of spirituality and community support in post‐transplant well‐being.

### 6.1 Relevance to Clinical Practice

This study underscores the critical need for nurses to conduct comprehensive, holistic comfort assessments, guided by Kolcaba's framework, to address the multifaceted needs of post‐transplant patients. Clinically, interventions should be tailored to identified physical (e.g., pain management), environmental, socio‐financial (e.g., connecting to resources) and psychospiritual needs. Institutionally, these findings advocate for enhancing clinical environments, integrating robust multidisciplinary support (including psychological and social services) and improving access to financial aid to truly promote patient well‐being.

For future research, we suggest exploring the influence of support networks and religion more deeply on transplant patients’ comfort perceptions. Longitudinal studies are also crucial to understand how comfort needs evolve over time after transplantation.

## Author Contributions

**Cecília Carla Barroso Calazans** and **Jennara Cândido do Nascimento:** conceptualization, investigation, formal analysis, methodology, data curation, supervision, writing – original draft, writing – review and editing. **Renan Alves da silva** and **Francisco Gilberto Fernandes Pereira:** methodology, writing – review and editing. **Lívia Moreira Barros** and **Joselany Áfio Caetano:** investigation, formal analysis, writing – review and editing. All authors participated sufficiently in the work to take public responsibility for appropriate portions of the content and agreed to be accountable for all aspects of the work.

## Funding

The authors have nothing to report.

## Conflicts of Interest

The authors declare no conflicts of interest.

## Supporting information


**Data S1:** Supporting Information 1. Interview guide.

**Data S2:** COREQ (Consolidated criteria for Reporting Qualitative research) Checklist.

## Data Availability

The data that support the findings of this study are available from the corresponding author upon reasonable request.
