# Supplementary material for: Comfort Needs of Renal Transplant Recipients: A Qualitative Analysis Guided by Kolcaba's Theory of Comfort
Source: J Clin Nurs. 2026 Jan 2;35(5):2496–509. doi: 10.1111/jocn.70188 (PMC13068178; doi:10.1111/jocn.70188)
Supplement: Supplementary file 1 — Data S1: Supporting Information 1. Interview guide. [file JOCN-35-2496-s002.docx]

**Supplementary Material 1.** Interview guide

**BASIC INTERVIEW INFORMATION**

**Time: ___:___ Date:** _____/_____/_____

**Interview duration:** _____:_____

Location where the interview took place:______________________________________

Interviewer's name:___________________________________________________

Interviewee's name:____________________________________________________

Audio recording file code:_________

**INITIAL PROCEDURES**

- Introduction of the researcher and the objectives of the interview.
- Reinforce confidentiality and the freedom to withdraw at any time.
- Request consent to record the interview.
- Ask the opening question: "To begin, I'd like you to talk a little about your experience after the kidney transplant. What has changed in your life since the transplant?"

**INTERVIEW CONTENT QUESTIONS**

1. How do you perceive and understand the concept of 'comfort' in your daily life?
2. Describe the feelings or situations that cause you discomfort after your kidney transplant.
3. For you, what were the main health needs after the kidney transplant? How did meeting those needs help (or how do they continue to help) you feel more comfortable during this period?

**CLOSING PROCEDURES**

- Thank the interviewee for their time and willingness to participate in the interview.
- Answer any questions and/or doubts that may arise.
- Reiterate the confidentiality of the interview to the interviewee.
- Ask if they would be willing to be interviewed again in case any points need clarification.
